# *Bacillus halotolerans* XJ-1 inhibits the fungal pathogen *Fusarium asiaticum* while promoting the growth of maize

**DOI:** 10.1038/s41598-026-55498-6

**Published:** 2026-05-28

**Authors:** Xiaohua Hao, Yating Dong, Jianming Wang

**Affiliations:** 1 College of Plant Protection, Shanxi Agricultural University, Taigu, 030800 Shanxi China; 2 Department of Life Sciences, Xinzhou Normal University, Xinzhou, 034000 Shanxi China

**Keywords:** *Bacillus halotolerans*, *F. asiaticum*, Growth indicators, Physiological indicators, Plant physiology, Plant stress responses

## Abstract

This study explored the potential of *Bacillus halotolerans* XJ-1 as a biocontrol agent against *Fusarium asiaticum*, the pathogen of maize stem base rot. Pot experiments were conducted to determine the morphological and physiological indicators of corn treated with and without strain XJ-1, and the incidence rate, disease index, and relative control efficacy were calculated. The effects of the fermentation filtrate of XJ-1 on the reactive oxygen species content and the activities of related defense enzymes of *F. asiaticum* were determined. Compared with the treatment of only inoculating *F. asiaticum*, only inoculating XJ-1 for biological enhancement significantly alleviated the severity of maize stem base rot and reduced the disease incidence. The protective and therapeutic efficacy of XJ-1 against *F. asiaticum* was 61.7% and 56.3%, respectively. Compared with the control, XJ-1 significantly promoted the growth of corn with an increase of plant height, stem diameter, dry and fresh weight of leaves, leaf length, leaf width, and leaf area by 32.30%, 33.33%, 24.48%, 32.14%, 40.66%, 20.92%, and 28.95%, respectively. The contents of chlorophyll, soluble protein, soluble sugar, nitrate nitrogen, and inorganic phosphorus in the leaves all increased significantly by 1.75, 1.29, 1.07, 1.47, and 1.40 times that of the control, respectively. The fermentation filtrate of XJ-1 significantly increased the contents of hydrogen peroxide and superoxide anion, along with the clearance rate of the hydroxyl radical, whereas the activities of superoxide dismutase, peroxidase, polyphenol oxidase, and phenylalanine ammonia-lyase were significantly reduced. Moreover, XJ-1destroyed the hyphae and cell structure of *F. asiaticum*, leading to depression, dryness, uneven thickness, and leakage of cell contents. Therefore, strain XJ-1 has a significant promoting effect on corn plants suffering from corn stem base rot with potential application value in biological control and agricultural production.

## Introduction

Maize is one of the most important agricultural crops worldwide. Owing to its advantages of low planting cost, abundant nutrients, and high comprehensive development and utilization value, maize is considered a “golden industry” in the agricultural economy. In recent years, there has been a rising incidence of corn diseases driven by several factors, including increased limitation of the corn planting environment, changes of climate factors, outdated cultivation management modes, greater continuous cropping areas, promotion of *Platycodon grandiflorum* returning technology, flawed agronomic measures, and the long growth cycle, contributing to a continuous accumulation of pathogenic bacteria in the field, thereby restricting the sustainable development of the corn industry^1^. Corn stalk rot has emerged as an important soil-borne disease of maize, with high incidence and wide distribution in China. Corn stalk rot, also known as corn bacterial wilt, causes both root and stem base rot in corn, characterized by early infection, late onset, and infection at various stages of growth. This disease is mainly caused by the single or compound infection of *Fusarium* species, and symptoms appear during the corn filling stage, with the peak of symptoms occurring from the late milk-ripening stage to the wax-ripening stage. The main symptoms include a withered and wilted stem; hot, dry, and faded leaves; dead bracts; drooping ears; a water-soaked stem base; hollow feeling when pinched by hand; and ease in lodging of the stem. Therefore, corn stem rot is resulting in a significant reduction in maize yield^2^.

*Fusarium asiaticum* is an important plant pathogen fungal species in the Tuberculariaceae family that can cause severe diseases in various crops such as wheat, barley, and corn. Its pathological effects are significant, often leading to scab, stem rot, and ear rot, thereby posing a significant threat to the agricultural economy. Given its economic importance, the pathogenic mechanisms and related impacts of *F. asiaticum* have been extensively studied^3–5^. *F. asiaticum* can also produce a variety of mycotoxins such as deoxynivalenol, which not only endangers the health of crops but may also threaten the safety of humans and animals through the food chain^6,7^. *F. asiaticum* is the causal pathogen of the epidemic of corn stem base rot spreading widely across China^8^. Its severity now exceeds that of smut, corn large spot, and corn small spot, becoming the disease of primary concern in agricultural production. At present, disease control mainly relies on the use of chemical agents. However, long-term application of chemicals not only induces the development of drug resistance in pathogens but also aggravates environmental pollution problems^9–11^. Therefore, developing green and environmentally friendly biological control strategies has become an important research focus.

*Bacillus halotolerans* is a bacterial species demonstrated to have inhibitory effects on numerous plant pathogens, indicating its significant application value in biological control^12,13^. *B. halotolerans* has inhibitory effects on the growth of various plant pathogens and its secondary metabolites can also kill or inhibit the germination of pathogen spores, destroy the cell structure of pathogens, and promote plant growth. *B. halotolerans* YP2 was isolated by Sarmah et al.^14^ from the rhizosphere soil of coastal halophytes, which was shown to enhance the salt resistance of oats and promote their growth, reduce the content of malondialdehyde in oat leaves, and increase the activity of antioxidant enzymes. Mondol et al.^15^ found that *B. halotolerans* QTH8can inhibit the growth of the pathogenic fungus *Fusarium oxysporum*, causing severe deformation of the hyphae, and exhibited killing activity against the second-instar larvae of soybean cyst nematodes, offering a candidate biological agent for the prevention and control of wheat stem rot and soybean cyst nematodes. Strain QTH8 also exhibited broad-spectrum antimicrobial activity. The culture filtrate of strain QTH8 showed antagonistic activity against various pathogenic fungi, including *Fusarium solani*, *Fusarium graminearum*, and *Fusarium oxysporum*. Strain QTH8 further demonstrates robust environmental stability with insensitivity to low temperatures and ultraviolet radiation, and its antibacterial activity was not substantially affected under harsh conditions, suggesting strong environmental adaptability. Moreover, Zhou et al.^16^ found that *B. halotolerans* S7 exhibited extremely strong β-1,4-endoxylanase activity. When applied to the hydrolysis of agricultural waste (corn cob and hardwood pulp), this strain efficiently obtained high-value oligosaccharides. The β -1,4-endoxylanase activity in *B. halotolerans* S7 was further improved through isothermal compression perturbation engineering and computer-aided design combined with three thermal stability prediction algorithms. This process resulted in the mutant strain R69F, which showed the most significant improvement in specific activity, heat resistance, and alkali resistance: its specific activity increased by 4.9 times and the temperature half-life increased by 39.4 times. Therefore, substantial research to date has shown that *B. halotolerans* has broad-spectrum antibacterial activity, can inhibit the growth of pathogenic microorganisms, break the mycelial structure of pathogenic fungi, activate the plant defense system, improve the activity of plant defense enzymes, and enhance plant disease resistance. Importantly, these traits can also be enhanced by targeted mutation or genetic modification to obtain more practical engineering strains for production, offering great potential for development as biocontrol and engineering bacteria.

Recent studies have further revealed that *B. halotolerans* strains can actively promote the growth and development process of plants^17^. In particular, *B. halotolerans* BY-S2 exhibited excellent resistance against the tobacco pathogen *Phytophthora nicotianae*^18–20^. This strain dissolves phosphorus and can also produce iron carriers, demonstrating good tolerance in an environment contaminated with the heavy metal copper^21,22^. Although the antagonistic mechanism of other biocontrol bacteria against *F. asiaticum* has been preliminarily explored, such as *Streptomyces* WY3^23–25^, the potential inhibitory effect of *B. halotolerans* against *F. asiaticum* and its growth-promoting effect on maize have not yet been clarified.

To address this gap, in this study, we evaluated the potential inhibitory effect of *B. halotolerans* XJ-1 on *F. asiaticum* along with its promoting effect on growth indicators and physiological and biochemical characteristics of maize plants. This study can therefore provide a theoretical basis and scientific support for the green prevention and control of corn stem base rot.

## Materials and methods

### Bacterial strain and culture

*B. halotolerans* XJ-1 was isolated from healthy corn plants and stored in the Biochemistry Laboratory of Xinzhou Normal University. Before use, the bacterium was activated at 28 °C on nutrient Luria–Bertani (LB) medium (10 g peptone, 5 g yeast extract, 10 g sodium chloride, 1000 mL distilled water) with shaking at 180 rpm for 12 h. The seed solution was obtained and stored for future use. The seed liquid was then inoculated into LB medium and oscillated at 28 °C and 180 rpm for 72 h to obtain the fermentation broth of strain XJ-1^26^. The fermentation broth was then centrifuged at 8000 rpm for 20 min, and the supernatant was collected and filtered through a 0.22-µm microporous filter membrane to obtain the final fermentation filtrate of XJ-1^27^.

### Fungal strain and culture

*F. asiaticum* was isolated from plants affected by maize stem base rot. The strain was stored in the Biochemistry Laboratory of the Biology Department of Xinzhou Normal University. Before use, the strain was activated at 25 °C on potato dextrose agar (PDA) medium (200 g potato, 20 g glucose, 18 g agar, 1000 mL distilled water) for 7 days, referring to the method of Lachhab et al.^28^. To prepare the spore suspension, 10 mL of sterile water was added to the plate along with two drops of Tween-80. The spores were shaken well, scraped off the PDA plate, and transferred to a conical flask. The suspension was filtered with four layers of gauze and diluted to 1 × 10^6^ spores/mL using a hemocytometry plate. The final suspension was stored at 4 °C for future use. To obtain mycelia, the *F. asiaticum* spore suspension was added to LB medium and cultured with shaking at 25 °C and 160 rpm for 48 h. The mycelium (0.5 g) was added to LB medium containing 50% of the XJ-1 fermentation filtrate by volume fraction for 36 h of culture. The mycelia were collected at 0, 3, 6, 9, 12, 24, and 36 h of culture; washed with sterile water three times; and stored in liquid nitrogen for future use. Sterile water (CK) served as the control for inoculation experiments.

### Plant growth experiments

Plant growth experiments were conducted with the maize variety Xianyu 1620 (disease-free), purchased from the Maize Institute of Shanxi Academy of Agricultural Sciences.Pot experiments were established at the Biochemistry Laboratory of the Biology Department in Xinzhou Normal University.

Plump corn seeds were selected, disinfected with a 0.1% sodium hypochlorite solution for 10 min, rinsed with distilled water, soaked for 12 h, and left to germinate for 3 days in a 20-cm-diameter culture dish with two layers of filter paper at 28 °C. The corn seedlings that exhibited uniform growthwere selected and transferred to a seedling culture box in an artificial climate chamber (day/night temperature of 28 °C/25°C, day/night cycle of 16 h/8 h, light intensity of 12,000 lx, relative humidity of 65%) for cultivation. When the corn reached the three-leaf and one-heart stage, seedlings with similar growth were selected for treatment with three biological replicates established for each condition.

A total of six treatments were set up with 16 plants per group: (1) single inoculation of XJ-1 (XJ-1 group), (2) single inoculation of *F. asiaticum* (FA group), (3) inoculation of XJ-1 first followed by inoculation of *F. asiaticum* 7 days later (XJ-1 + FA group), (4) inoculation of *F. asiaticum* first followed by XJ-1 treatment after disease onset (FA + XJ-1 group), (5) inoculation of *F. asiaticum* first followed by root irrigation with 50% carbendazim wettable powderas a positive control (FA + C group), and (6) inoculation with sterile water (CK group). Inoculation began on March 12, 2025. The disease status of the corn plants was observed every 4 days after treatment, and the incidence rate, disease index, and relative control effect were calculated and compared after 50 days, following the maize root rot grading standards described by Yin et al.^29^:

Incidence rate = (Number of affected plants/Total number of investigated plants) × 100%.

Disease index = [∑(Number of diseased plants at each level × disease grade)/(Total number of investigated plants × highest value)] × 100.

Relative prevention effect = [(control disease index – treatment disease index)/control disease index] × 100%.

### Inhibitory effect of XJ-1 on the growth of *F. asiaticum*

The fermentation supernatant (2 mL) of strain XJ-1 was mixed thoroughly with 20 mL of pre-prepared and high-temperature-sterilized PDA medium before pouring the plates. After solidification, a mycelial cake of *F. asiaticum* was inoculated in the center of the plate using the hole-punching method, with the mycelial side facing down, followed by incubation in a constant-temperature biochemical incubator at 25 °C. For the control group (CK), the fermentation supernatant of strain XJ-1 was replaced with an equal amount of sterile basal liquid medium while maintaining all other procedures identical. The colony diameter was measured on the 3rd, 5th, 7th, 9th, and 11th days of incubation using the cross-line method. Observations were stopped when the mycelium of *F. asiaticum* completely covered the control plate. Three replicate plates were established for each experimental group.

The fermentation filtrates of strain XJ-1 were mixed with PDA medium in volume fractions of 10%, 20%, 30%, 40%, 50%, 60%, and 70% and then poured into petri dishes. The activated *F. asiaticum* cake was placed in the center of the plate (diameter of the fungal plug was 5 mm) and the PDA plate without the fermentation filtrate of XJ-1 served as the control. The plates were cultured at 25 °C, with three replicates established for each treatment. Once *F. asiaticum* covered the entire control plate, the colony diameter was measured by the cross-line method and the mycelial growth inhibition rate was calculated using the following formula:

Inhibition rate = [(control colony diameter – treatment colony diameter)/(control colony diameter – 5 mm)] × 100%.

### Determination of maize morphological indicators and leaf physiological indicators

The fourth fully spread leaf at the top of the corn seedlings was selected and the four consecutive leaves were collected downward. The plant height (distance from the bottom of the stem to the top bud) and stem diameter of the corn plants were measured with a tape measure and a Vernier caliper, respectively. The leaf length, leaf width, and leaf area were determined by a leaf area meter. The fresh weight of the leaves was measured by an electronic balance. The plants were dried at 80 °C until the mass was constant. The same measurement method used for the underground parts of corn plants was adopted for the above-ground parts, using the straight-edge measurement method and oven-drying. The chlorophyll content was determined by the dimethyl sulfoxide extraction method, the soluble sugar content was determined by the anthrone colorimetric method, the soluble protein content was determined by the Coomassie brilliant blue G-250 method, the nitrate nitrogen content was determined by the salicylic acid method, and the inorganic phosphorus content was determined by the molybdenum blue method.

### Determination of reactive oxygen species (ROS) contents in *F. asiaticum*

The contents of H_2_O_2_ and O_2_ and the clearance rate of •OH in *F. asiaticum* were determined from a homogenate obtained by grinding 0.1 g of the *F. asiaticum* mycelium in 1 mL of extract using respective kits from Beijing Solarbio Technology Co., Ltd., with item numbers BC3590, BC1290, and BC1320, respectively.

For the determination of H_2_O_2_ content, after centrifuging the homogenate at 4 °C and 8000 rpm for 10 min, the supernatant was added to the sample according to the determination steps of the kit. The absorbance value was measured at 415 nm with a spectrophotometer and used to calculate the H_2_O_2_ content (µmol/g). For the determination of O_2_^−^ content, the homogenate was centrifuged at 4 °C and 12,000 rpm for 20 min. The absorbance value of the supernatant was measured at 530 nm and used to determine the O_2_^−^content (µmol/g) according to the standard curve.For determination of the •OH clearance rate, the homogenate was centrifuged at 4 °C and 10,000 ×*g* for 10 min. The absorbance of the supernatant at 536 nm was measured and used to calculate the •OH clearance rate (%) based on the kit instructions.

### Determination of the activities of *F. asiaticum* defense-related enzymes

The activities of superoxide dismutase (SOD), peroxidase (POD), polyphenol oxidase (PPO), and phenylalanine ammonia-lyase (PAL) were determined from a homogenate obtained by grinding 0.1 g–0.05 g of the *F. asiaticum* mycelium in 1 mL of extract using respective kits from Beijing Solarbio Technology Co., Ltd., with item numbers BC0200, BC0090, BC0170, and BC0220, respectively.

For SOD activity determination, the homogenate was centrifuged at 4 °C and 8000 ×*g* for 10 min. The absorbance value of the supernatant at 560 nm was determined and the SOD activity (U/g) was calculated according to the kit instructions.

For POD activity determination, the homogenate was centrifuged at 8000 ×*g* at 4 °C for 10 min and the supernatant was collected. The absorbance values of the supernatant were measured at 470 nm at 30 and 90 s according to the kit instructions to calculate the POD activity (U/g).

For PPO activity determination, the homogenate was centrifuged at 4 °C and 13,000 ×*g* for 20 min and the supernatant was collected. The absorbance value of the supernatant was measured at 425 nm to calculate the PPO activity (U/g) according to the kit instructions.

For PAL activity determination, the homogenate was centrifuged at 4 °C and 5000 rpm for 10 min. The absorbance value of the supernatant at 290 nm was measured and the PAL activity (U/g) was calculated according to the kit instructions.

### Effect of strain XJ-1 on the mycelial morphology of *F. asiaticum*

The spore suspension of *F. asiaticum* was diluted to 1 × 10^6^ spores/mL and then inoculated into 50mL PDB medium at a 4% inoculation rate. After cultivation on a shaker at 28° C and 150 rpm for 48 h, a 10% inoculum of the 6-fold-concentrated supernatant of strain XJ-1 was added to the PDB medium after 2 days of cultivation as the treatment group. The control group comprised an equal amount of sterile water rather than strain XJ-1. Both groups were cultured separately at 28° C with shaking at 150 rpm. Subsequently, the mycelia of the treatment and the control groups were collected and observed under a scanning electron microscope.

The mycelia were rinsed three times with 10mL phosphate-buffered saline (PBS) for 20 min each time and fixed in a culture dish with 2.5% glutaraldehyde, corresponding to 20 times the volume of the sample. After refrigerating the samples at 4° C overnight, the mycelium was separated from the fixative and thoroughly rinsed with PBS three times for 15 min each time. Subsequently, the mycelial samples were soaked in a 1% osmium oxide solution for fixation for 2 h. The fixative was discarded and the samples were washed three times with PBS for 15 min each time. The mycelium was placed in ethanol solutions with different concentration gradients of 50%, 70%, 80%, 90%, and 95%for 10 min at each concentration. The mycelium was soaked and dehydrated twice in anhydrous ethanol for 15 min. Finally, the mycelium was placed on a glass slide in a culture dish, air-dried in a dryer for 4 h, coated, and observed under the scanning electron microscope.

### Statistical analysis

Experimental data were processed using Microsoft Excel 2022. SPSS 27.0 was used to determine the statistical significance of differences between groups with analysis of variance and Duncan’s method for multiple comparisons. Statistical significance was judged at the *P* < 0.05 level. Origin 2022 software was used for plotting.

## Results

### Control effect of *B. halotolerans* XJ-1 on maize stem base rot

**Fig. 1:**
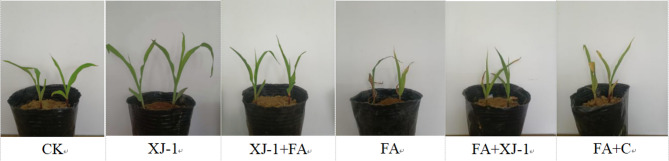
Effect of *Bacillus halotolerans* XJ-1 on maize stem base rot in the pot experiment. CK, control (inoculated with sterile water); XJ-1, single inoculation of *Bacillus halotolerans* XJ-1; FA, single inoculation of *F. asiaticum*; XJ-1 + FA. inoculated first with *Bacillus halotolerans* XJ-1 and then with *F. asiaticum* 7 days later; FA + XJ-1, inoculated with *F. asiaticum* first and then with *Bacillus halotolerans* XJ-1 after disease occurrence; FA + C, inoculated with *F. asiaticum* first and then the roots were applied a 50% carbendazim wettable powder after disease occurrence.

The indices used to examine the control effect of strain XJ-1 on maize stem base rot are presented in Table 1 with representative photographs shown in Fig. 1.

**Table 1:** Control effect of *Bacillus halotolerans* XJ-1 on maize stem base rot.

Treatment	Incidence rate (%)	Disease index	Relative control effect (%)
CK	31.4 ± 1.03	9.8 ± 0.02	-
XJ-1	16.8 ± 0.16	7.6 ± 0.01	-
XJ-1 + FA	24.7 ± 0.24	10.6 ± 0.05	61.7 ± 0.32
FA	63.8 ± 1.52	31.3 ± 0.04	-
FA + XJ-1	25.7 ± 0.63	12.62 ± 0.11	56.3 ± 0.12
FA + C	37.4 ± 0.32	18.31 ± 0.64	41.9 ± 0.09

CK, control (inoculated with sterile water); XJ-1, single inoculation of *Bacillus halotolerans* XJ-1; FA, single inoculation of *F. asiaticum*; XJ-1 + FA, inoculated first with *Bacillus halotolerans* XJ-1 and then with *F. asiaticum* 7 days later; FA + XJ-1, inoculated with *F. asiaticum* first and then with *Bacillus halotolerans* XJ-1 after disease occurrence; FA + C, inoculated with *F. asiaticum* first and then the roots were applied a 50% carbendazim wettable powder after disease occurrence.

As shown in Table 1, the maize inoculated with *F. asiaticum* had the highest incidence of stem base rot (63.8%), which was drastically decreased with pre-inoculation of *B. halotolerans* XJ-1 for 7 days (24.7%); the relative control effect reached 61.7%. The incidence of corn stem base rot was the lowest (16.8%) in corn inoculated only with *B. halotolerans* XJ-1. The incidence of corn stem base rot after inoculation with *F. asiaticum* and subsequent inoculation with *B. halotolerans* XJ-1 was 25.7%, and the relative control efficacy reached 56.3%. This was a significantly higher efficacy than achieved using root irrigation with 50% carbendazim wettable powder of(41.9%).

Figure 1 shows that after 50 days of treatment, the corn inoculated with XJ-1 had the best growth, the most luxuriant leaves, and the largest leaf area. When the corn was first inoculated with XJ-1 followed by inoculation of *F. asiaticum* 7 days later, the plant grew well and had many leaves. The corn inoculated only with *F. asiaticum* exhibited poor growth, with yellow and more withered leaves.

**Table 2:** Effects of *Bacillus halotolerans* XJ-1 on the above-ground parts of maize.

Treatment	Plant height (cm)	Stem diameter (cm)	Fresh leaf weight (g)	Leaf dry weight (g)
CK	16.1 ± 1.67c	0.57 ± 0.009c	1.43 ± 0.036b	0.28 ± 0.006c
XJ-1	21.3 ± 2.68a	0.76 ± 0.005a	1.78 ± 0.043a	0.37 ± 0.009a
XJ-1 + FA	19.2 ± 2.04b	0.63 ± 0.008b	1.35 ± 0.051c	0.31 ± 0.007b
FA	8.4 ± 0.53 d	0.42 ± 0.005f	0.85 ± 0.027f	0.13 ± 0.003f
FA + XJ-1	15.8 ± 1.43 c	0.51 ± 0.010e	1.16 ± 0.031d	0.22 ± 0.004d
FA + C	16.0 ± 1.92 c	0.53 ± 0.006d	1.07 ± 0.033e	0.19 ± 0.006e

Treatment	Leaf length (cm)	Leaf width (cm)	Leaf area (cm^2^)	Leaf shape index
CK	18.2 ± 2.31 bc	1.53 ± 0.012c	52.98 ± 4.323 bc	11.895 ± 1.84 c
XJ-1	25.6 ± 2.78a	1.85 ± 0.023a	68.32 ± 3.282a	13.838 ± 2.32 bc
XJ-1 + FA	20.4 ± 3.31b	1.62 ± 0.031b	57.43 ± 3.972b	12.592 ± 2.17 bc
FA	11.5 ± 1.74 d	0.56 ± 0.042f	23.22 ± 4.172 d	20.536 ± 1.93 a
FA + XJ-1	15.8 ± 1.56 c	1.31 ± 0.021d	48.64 ± 2.901 c	12.061 ± 2.01 c
FA + C	17.9 ± 2.21 bc	1.24 ± 0.037e	46.68 ± 1.985 c	14.435 ± 1.72 b

CK, control (inoculated with sterile water); XJ-1, single inoculation of *Bacillus halotolerans* XJ-1; FA, single inoculation of *F. asiaticum*; XJ-1 + FA, inoculated first with *Bacillus halotolerans* XJ-1 and then with *F. asiaticum* 7 days later; FA + XJ-1, inoculated with *F. asiaticum* first and then with *Bacillus halotolerans* XJ-1 after disease occurrence; FA + C, inoculated with *F. asiaticum* first and then the roots were applied a 50% carbendazim wettable powder after disease occurrence.

Different lowercase letters indicate a statistically significant difference between groups (*P* < 0.05).

### Influence of *B. halotolerans* XJ-1 on the growth and morphological indicators of maize

The effects of strain XJ-1 on the growth and morphological indicators of corn are summarized in Table 2. The plant height significantly differed among treatment groups (*P* < 0.05). The plant height of corn treated with a single inoculation of XJ-1 fermentation broth was the largest, whereas the lowest plant height was detected in the corn treated with a single inoculation of *F. asiaticum*. Compared with the control (CK), a single inoculation of XJ-1 fermentation broth substantially increased the plant height, stem diameter, fresh and dry weights of the leaves, leaf length, leaf width, and leaf area of corn by 32.30%, 33.33%, 24.48%, 32.14%, 40.66%, 20.92%, and 28.95%, respectively. These results indicated that *B. halotoleran*s XJ-1 has a significant growth-promoting effect on corn.

Plant growth was also significantly better in the group with pretreatment of *B. halotolerans* XJ-1 7 days before *F. asiaticum* inoculation compared to that of plants inoculated only with *F. asiaticum.* Specifically, pretreatment with XJ-1 resulted in an increase in plant height, stem diameter, fresh weight of the leaves, dry leaf weight, leaf length, leaf width, and leaf area by 43.28%, 50.00%, 58.82%, 138.46%, 77.39%, 189.29%, and 147.33%, respectively. Similarly, treatment of XJ-1 after disease occurrence improved the growth indicators of corn compared with those of plants inoculated with *F. asiaticum* alone, with an increase of plant height, stem diameter, fresh leaf weight, dry leaf weight, leaf length, leaf width, and leaf area of 17.91%, 21.43%, 36.47%, 69.23%, 37.39%, 133.92%, and 109.47%, respectively. As a positive control, the growth parameters of the corn treated with 50% carbendazim wettable powder for root irrigation were also improved compared with those of the group only inoculated with *F. asiaticum*, with an increase of plant height, stem diameter, fresh leaf weight, dry leaf weight, leaf length, leaf width, and leaf area by 19.40%, 26.19%, 25.88%, 46.15%, and 55.65%, 121.43%, and 101.03%, respectively. Nevertheless, these improvements were slightly lower than those detected with pretreatment of *B. halotolerans* XJ-1. Therefore, corn treated with a single inoculation of *F. asiaticum* exhibited the weakest growth and *B. halotolerans* XJ-1 had a significant growth-promoting effect both prior to and following disease occurrence.

**Table 3:** Effects of *Bacillus halotolerans* XJ-1 on the underground parts of maize.

Treatment	Taproots number	Rootlength (cm)	Fresh weight of underground part (g)	Dry weight of underground part (g)
CK	4.00 ± 0.26ab	14.95 ± 1.17a	0.59 ± 0.02ab	0.17 ± 0.01a
FA	2.50 ± 0.30d	11.70 ± 0.98d	0.36 ± 0.01d	0.08 ± 0.01c
XJ-1	4.50 ± 0.22a	15.30 ± 1.04a	0.65 ± 0.04a	0.18 ± 0.02a
FA + XJ-1	3.50 ± 0.50b	13.70 ± 1.14ab	0.52 ± 0.03abc	0.12 ± 0.01b
XJ-1 + FA	3.60 ± 0.36b	13.53 ± 1.01ab	0.46 ± 0.02bc	0.11 ± 0.01b
FA + C	3.22 ± 0.42c	12.63 ± 0.21c	0.43 ± 0.13bc	0.10 ± 0.36b

CK, control (inoculated with sterile water); XJ-1, single inoculation of *Bacillus halotolerans* XJ-1; FA, single inoculation of *F. asiaticum*; XJ-1 + FA, inoculated first with *Bacillus halotolerans* XJ-1 and then with *F. asiaticum* 7 days later; FA + XJ-1, inoculated with *F. asiaticum* first and then with *Bacillus halotolerans* XJ-1 after disease occurrence; FA + C, inoculated with *F. asiaticum* first and then the roots were applied a 50% carbendazim wettable powder after disease occurrence.

Different lowercase letters indicate a statistically significant difference between groups (*P* < 0.05).

As shown in Table 3, compared to the control group, the XJ-1 treatment group exhibited significant increases in all indicators of underground parts, with improvements of 12.52%, 2.34%, 10.54%, and 5.10% for the number of roots, root length, fresh weight, and dry weight, respectively, indicating that strain XJ-1 promotes the growth of the underground parts of corn seedlings. The FA group showed slower growth and shorter root systems, with the above indicators respectively decreasing by 37.51%, 21.74%, 39.53%, and 49.97% compared to those of the control group, demonstrating that *F. asiaticum* significantly inhibits the growth of the underground parts of corn seedlings. Both the FA + XJ-1 and XJ-1 + FA treatment groups had lower indicators than those of CK but higher indicators than those of the FA treatment group. The FA + XJ-1 group showed increases of 40.22%, 17.09%, 47.33%, and 42.57% in taproots number, Root length, Fresh weight of underground part, Dry weight of underground part compared to those of the FA group, while the XJ-1 + FA group showed corresponding increases of 44.15%, 15.38%, 30.19%, and 30.11% compared to those of the FA group. The growth parameters of corn seedlings in the FA + C treatment group improved significantly compared to those of the FA treatment group, increasing by 22.36%, 7.95%, 19.44%, and 25.00%, but remained slightly lower than those of the CK. These findings indicated that strain XJ-1 not only significantly reduces the damage caused by *F. asiaticum* to the underground parts of corn seedlings but also results in better growth conditions for treated seedlings compared to the FA + C treatment, demonstrating effective defense against *F. asiaticum*-induced diseases.

**Table 4:** Effects of *Bacillus halotolerans* XJ-1 on the growth and morphological indicators of maize.

Treatment	Total chlorophyll (mg/g)	Soluble sugar content (%)	Soluble protein content (mg/g)	Nitrate nitrogen content (%)	Inorganic phosphorus content (mg/g)
CK	0.120 ± 0.003c	0.14 ± 0.007b	6.64 ± 0.011b	0.36 ± 0.006c	91.00 ± 4.021d
XJ-1	0.210 ± 0.002a	0.18 ± 0.003a	7.12 ± 0.024a	0.53 ± 0.007a	127.02 ± 2.510a
XJ-1 + FA	0.160 ± 0.003b	0.15 ± 0.014b	6.49 ± 0.031c	0.47 ± 0.013b	121.98 ± 3.287b
FA	0.033 ± 0.002f	0.07 ± 0.004e	5.51 ± 0.078 f	0.16 ± 0.004e	81.97 ± 2.800f
FA + XJ-1	0.060 ± 0.004e	0.12 ± 0.121c	5.97 ± 0.026d	0.32 ± 0.005d	94.98 ± 3.610c
FA + C	0.080 ± 0.001d	0.09 ± 0.008d	5.78 ± 0.045 e	0.34 ± 0.003c	86.04 ± 1.894e

CK, control (inoculated with sterile water); XJ-1, single inoculation of *Bacillus halotolerans* XJ-1; FA, single inoculation of *F. asiaticum*; XJ-1 + FA, inoculated first with *Bacillus halotolerans* XJ-1 and then with *F. asiaticum* 7 days later; FA + XJ-1, inoculated with *F. asiaticum* first and then with *Bacillus halotolerans* XJ-1 after disease occurrence; FA + C, inoculated with *F. asiaticum* first and then the roots were applied a 50% carbendazim wettable powder after disease occurrence.

Different lowercase letters indicate a statistically significant difference between groups (*P* < 0.05).

### Effect of *B. halotolerans* XJ-1 on the physiological indicators of maize

As shown in Table 4, compared with those of the CK treatment, the XJ-1 fermentation broth significantly increased the contents of total chlorophyll, soluble sugar, soluble protein, nitrate nitrogen, and inorganic phosphorus in maize leaves by 1.75, 1.29, 1.07, 1.47, and 1.40 times, respectively. Compared with the effects of a single inoculation with *F. asiaticum*, pretreatment with XJ-1 also significantly increased the contents of total chlorophyll, soluble sugar, soluble protein, nitrate nitrogen, and inorganic phosphorus by 4.85, 2.14, 1.18, 2.94, and 1.49 times, respectively. Similar effects were observed when corn was treated with XJ-1 after disease onset, with increases in the levels of total chlorophyll, soluble sugar, soluble protein, nitrate nitrogen, and inorganic phosphorus by 1.81, 1.71, 1.08, 2.00, and 1.16 times, respectively. Correspondingly, the treatment of 50% carbendazim wettable powder for root irrigation as a positive control showed increases of 2.42, 1.29, 1.05, 2.13, and 1.05 times in these same parameters, respectively, compared with those of plants treated with *F. asiaticum* alone. Therefore, the fermentation broth of strain XJ-1 not only promotes the growth of corn plants but also has good preventive and therapeutic effects on corn stem base rot.

### Inhibitory effect of *B. halotolerans* XJ-1 on *F. asiaticum*

**Fig. 2:**
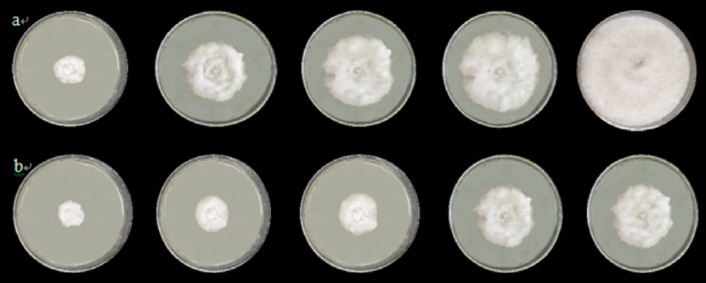
Inhibitory effect ofthe *Bacillus halotolerans* XJ-1 fermentation supernatant on the growth of*F. asiaticum* mycelia. a. Growth status of *F. asiaticum* on the sterile basic liquid medium (control group) at 3, 5, 7, 9, and 11 days.b. Growth of *F. asiaticum* in the supernatant of strain XJ-1 fermentation broth at 3, 5, 7, 9, and 11 days. The fermentation filtrates of strain XJ-1 were mixed with PDA medium in volume fractions of 10%, 20%, 30%, 40%, 50%, 60%, and 70% to prepare plates. The activated *F. asiaticum* cake was placed in the center of the plate and cultured at 25° C for 7 days. The colony diameter was measured by the cross-multiplication method and the inhibition rate was calculated.

The growth status of *F. asiaticum* on different treated media was determined use the cross-line method at 3, 5, 7, 9, and 11days of incubation. As shown in Fig. 2; Table 5, the growth rate of *F. asiaticum* on the strain XJ-1 fermentation supernatant medium was significantly lower than that detected when incubated on the control plate(in which the strain XJ-1 fermentation supernatant was replaced with an equal amount of water in the culture medium), demonstrating that *B. halotolerans* XJ-1 significantly inhibited the growth rate of *F. asiaticum*.

**Table 5:** Inhibitory effects of *Bacillus halotolerans* XJ-1 fermentation supernatant on mycelial growth of *F. asiaticum*.

Treatment	Days	Colony diameter (cm)	Inhibition rate (%)
CK	3 d	1.85 ± 0.01e	-
	5 d	3.42 ± 0.03d	-
	7d	5.70 ± 0.08c	-
	9 d	7.72 ± 0.04b	-
	11 d	8.84 ± 0.04a	-
Fermentation solution supernatant treatment	3 d	0.95 ± 0.01e	48.64 ± 0.54e
	5 d	1.50 ± 0.01d	56.14 ± 0.29d
	7 d	1.75 ± 0.02c	69.30 ± 0.35a
	9 d	3.65 ± 0.03b	52.72 ± 0.44b
	11d	4.57 ± 0.01a	48.30 ± 0.11c

As shown in Table 5, the inhibition rate of the fermentation supernatant of *B. halotolerans* XJ-1 on the mycelial growth of*F. asiaticum* showed a trend of first increasing and then decreasing. On the 7th day of cultivation, the highest inhibition rate reached 69.30% and then slightly decreased. This suggests that the *B. halotolerans* XJ-1 culture contains some secondary metabolites of microorganisms or substances with antimicrobial activity, which damage the growth points of *F. asiaticum*, cause hyphal dissolution, and inhibit the growth of *F. asiaticum*. In the early stage of cultivation, the antimicrobial substances in the treatment group culture medium have high activity and thus a significant antimicrobial effect. In the later stage of cultivation, the antimicrobial substances in the culture medium are affected by the surrounding environment or pH value, and the antibacterial activity slightly decreases, resulting in a decrease in the antimicrobial rate.

**Fig. 3:**
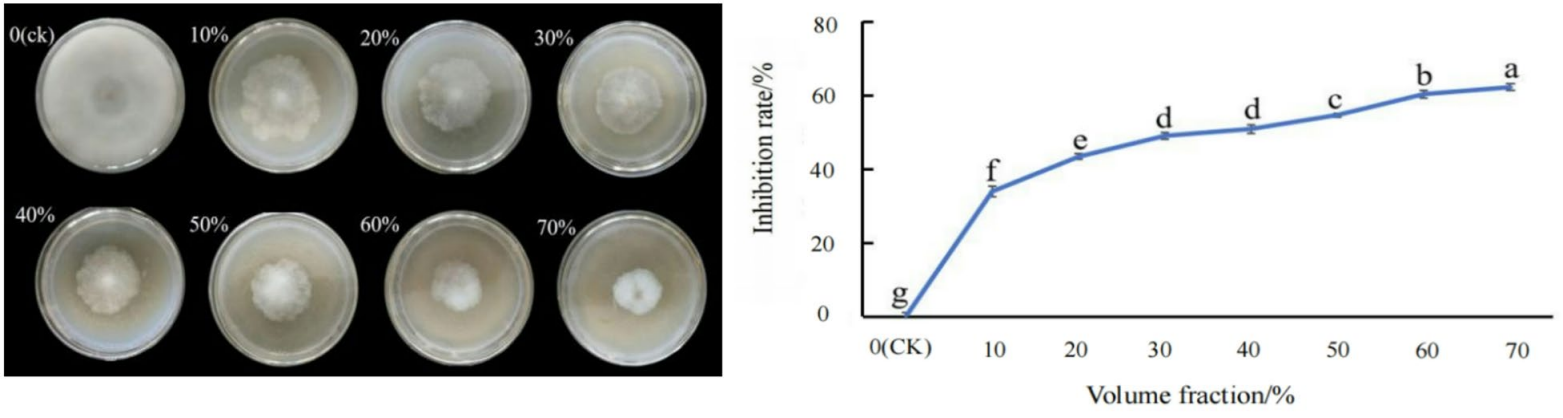
Inhibitory effect of *Bacillus halotolerans* XJ-1 on *Fusarium asiaticum*. Different lowercase letters indicate significant differences among different treatments (*P* < 0.05).

Figure 3 shows that the inhibition rate of XJ-1 on *F. asiaticum* increased significantly with the increase of the volume fraction of the XJ-1 fermentation filtrate to the PDA medium. XJ-1 had the strongest inhibitory effect on *F. asiaticum* when the volume fraction was 70%, with an inhibition rate of 71.11%, while the lowest inhibition rate (37.50%) was obtained with a volume fraction of 10%. When the volume fractions were 20%, 30%, 40%, 50%, and 60%, the inhibition rates of the XJ-1 fermentation filtrate against *F. asiaticum* were 44.44%, 55.56%, 59.82%, 62.26%, and 64.42%, respectively. There was no significant difference in the inhibition rates detected between the 50% and 60% volume fractions. Based on these findings, subsequent experiments were conducted using assessments on the 7th cultivation day with the 50% volume fraction fermentation broth of XJ-1.

**Fig. 4:**
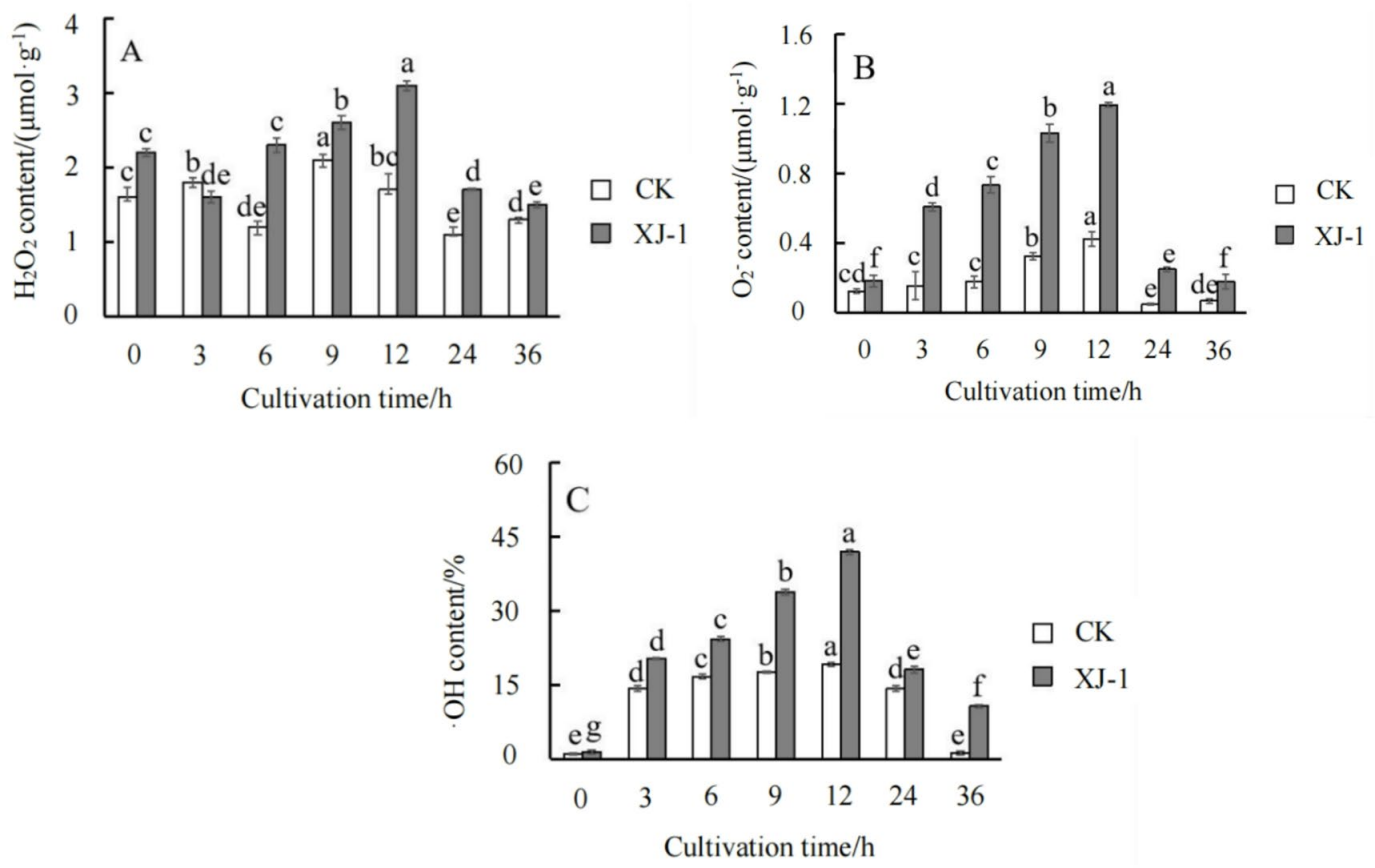
Effect of *Bacillus halotolerans* XJ-1 on the reactive oxygen species contents of *F. asiaticum*: (**A**) H_2_O_2_, (**B**) O_2_^−^ (superoxide anion), (**C**) •OH (hydroxyl radical). Different lowercase letters above the bars indicate significant differences (*P* < 0.05) for the same treatment at different incubation times.

### Effect of *B. halotolerans* XJ-1 on the ROS contents of *F. asiaticum*

As shown in Fig. 4, with the extension of culture time, the contents of H_2_O_2_ and O_2_^−•^ and the clearance rate of •OH of *F. asiaticum* showed an overall trend of first increasing and then decreasing. After treatment with XJ-1, all three ROS metrics were significantly higher than those of the control (*P* < 0.05), which reached their respective peaks at 12 h of culture. After treatment with XJ-1, the H_2_O_2_ content of *F. asiaticum* at 12 h of culture was 3.1 µmol/g, which was 1.82 times that of the control (Fig. 4A). These results indicated that XJ-1 has a certain stimulating effect on *F. asiaticum* to significantly promote the increase of H_2_O_2_ content.

Similarly, after culture for 12 h, the O_2_^−•^ content of *F. asiaticum* treated with XJ-1 was 1.193 µmol/g, which was 2.82 times that of the control (Fig. 4B). After culture for 12 h, the •OH clearance rate of *F. asiaticum* treated with XJ-1 reached the maximum value of 41.9%, which was 2.18 times that of the control (Fig. 4C).

**Fig. 5:**
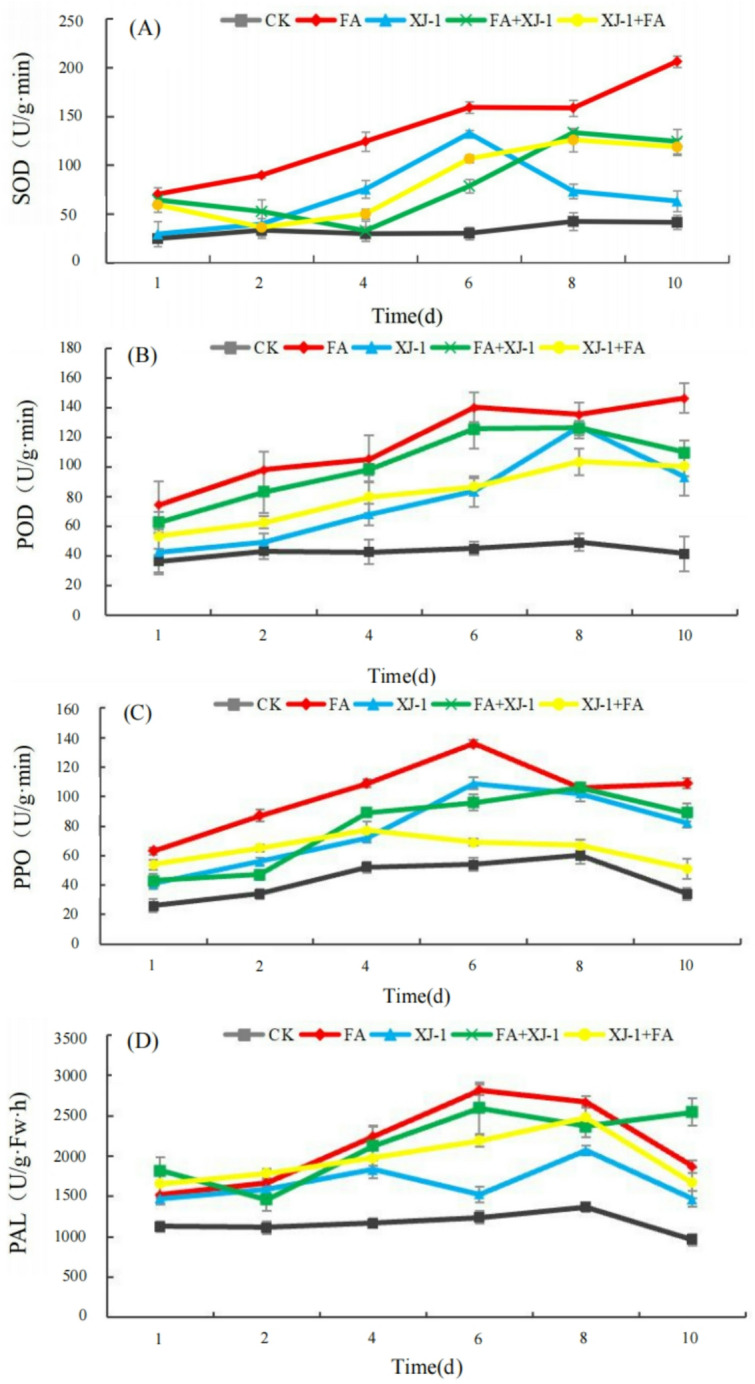
Effect of *Bacillus halotolerans* XJ-1 on the activities of *Fusarium asiaticum* defense-related enzymes: (**A**) superoxide dismutase (SOD), (**B**) peroxidase (POD), (**C**) polyphenol oxidase (PPO), and (D) phenylalanine ammonia-lyase (PAL). Different lowercase letters above the bars indicate significant differences (*P* < 0.05) for the same treatment at different incubation times.

### Effect of *B. halotolerans* XJ-1 on the activities of *F. asiaticum* defense-related enzymes

As shown in Fig. 5A, SOD enzyme activity in the CK treatment group remained stable during the first 10 days of growth in the corn three-leaf stage, which was consistently lower than that of the other treatment groups. With XJ-1 treatment, the enzyme activity initially increased and then decreased over 1–10 days, closely matching the activity levels of the control group at 1–2 days. The enzyme activity reached its peak at 132.62 U/g·min on day 6 and declined to 103.11 U/g·min by day 10. Despite this decrease over time, the enzyme activity remained significantly higher than that of the CK group, indicating that strain XJ-1 activated the corn plant’s defense system to enhance its resistance. In the FA treatment group, SOD enzyme activity continuously increased over days 1–10 and was significantly higher than that of the blank control and other treatment groups, peaking at 206.32 U/g·min on day 10. This suggests that pathogen invasion triggered a strong defense response in the plant, leading to a sharp rise in enzyme activity to resist pathogen damage and protect the plant. In the FA + XJ-1 treatment group, SOD enzyme activity slightly decreased during 1–4 days before sharply increasing to a peak of 133.43 U/g·min on day 8. SOD enzyme activity in the FA + XJ-1group was significantly lower than that of the FA group throughout the experimental period (1–10 days). The SOD enzyme activity in XJ-1 + FA treatment group was largely consistent with that in the FA + XJ-1 group. Both treatment combinations effectively suppressed pathogen damage to corn plants to varying degrees, thereby mitigating disease occurrence.

Figure 5B shows that during the first 10 days of the corn three-leaf stage, POD enzyme activity in the CK group remained stable without significant changes and was lower than that of the other treatment groups, indicating that the CK group was not infected by an external pathogen or non-pathogenic colonizer during the growth process. The enzyme activity of the XJ-1 treatment group showed an overall trend of a slow increase followed by a decrease from day 1 to day 10; the POD activity reached its highest value of 127.35U/g·min on the 8th day and then decreased to 93.13U/g·min on the 10th day. Within 2 days, it decreased by 26.85%, indicating that under the action of strain XJ-1, maize plants first developed induced resistance to improve their defense ability and the enzyme activity decreased after the plants adapted to the growth environment in the later stage. The POD enzyme activity in the FA treatment group showed an overall upward trend within 1–10 days, with significantly higher enzyme activity values than found for the CK group and other groups. At 10 days, POD enzyme activity reached its highest value of 146.31U/g·min, which was 3.54 times higher than that of the CK group at the same time. This indicates that after the pathogen infects the plant, it causes a strong defense response, leading to a sustained increase in POD enzyme activity. The enzyme activity of the FA + XJ-1 group slowly increased from 1 to 6 days, reaching a maximum value of 125.62U/g·min at 6 days. The POD enzyme activity of the FA + XJ-1 group was significantly lower than that of the FA group and was higher than that of the CK group for the 10-day observation period. This may be due to the presence of the pathogenin the mixed solution, causing damage to the growth of maize plants, leading to an increase in enzyme activity. However, the inhibitory effect of strain XJ-1 on the pathogenresulted in a lower increase in enzyme activity than that of the FA group. The overall enzyme activity of the XJ-1 + FA treatment group was lower than that of the FA + XJ-1 group, indicating that the inoculation of strain XJ-1 had a pre-protective effect on the plants, reducing the damage of pathogens that were subsequently inoculated on the plants.

As shown in Fig. 5C, PPO enzyme activity was the lowest in the CK treatment group among all treatments, slowly increasing from days 1 to 8 and then slightly decreasing after 8 days. The overall level of PPO enzyme activity in the XJ-1 treatment group was higher than that of the CKgroup, reaching its highest point at 6 days and then slowly decreasing thereafter. In the FA treatment group, PPO enzyme activity first continued to increase and then slowly decreased, stabilizing after 8 days and reaching a maximum value of 136.21U/g·min at 6 days. Within 1–10 days, PPO enzyme activity was higher than that of the CK group and other treatment groups, indicating that the invasion of *F. asiaticum* on maize seedlings caused significant damage to plant growth, leading to a rapid increase in PPO enzyme activity to defend against pathogen invasion and induce maize plants to develop acquired systemic resistance. The FA + XJ-1 treatment group showed a slow increase in enzyme activity from 1 to 8 days, reaching its highest point at 8 days. The overall enzyme activity level was lower than that of the FA group and higher than that of the other treatments. In the XJ-1 + FA treatment group, the enzyme activity showed a slow upward trend from 1 to 4 days; at 4 days, the enzyme activity reached a small peak of 77.23 U/g·min, decreasing to 51.41 U/g·min at 10 days, which was lower than the activity levels of the FA and FA + XJ-1groups. Therefore, it can be inferred that with the invasion of *F. asiaticum* on plants, strain XJ-1 is in a confrontational state with *F. asiaticum*, inducing systemic resistance in plants. The level of the natural PPO enzyme activity increases, which then decreases with enhancement of the inhibitory effect of strain XJ-1 on *F. asiaticum*, alleviating the excessive defense response of plants and protecting them from injury.

As shown in Fig. 5D, PAL enzyme activity in the leaves of the CK group changed from 1130 U/g·Fw·h to 968 U/g·Fw·h during the measurement period and remained stable overall. The PAL enzyme activity of the XJ-1 treatment group slowly increased to 1836U/g·Fw·h from 1 to 4 days, decreased to 1530U/g·Fw·h at 6 days, reached its highest value of 2071U/g·Fw·h on 8 days, and then decreased to 1468U/g·Fw·h on 10 days, with slight fluctuations overall. It is speculated that this was due to the increased secretion of PAL enzyme by maize seedlings under the action of strain XJ-1 to enhance their own defense ability. The PAL enzyme activity of the FA treatment group showed an overall trend of first increasing and then decreasing, with little change detected within 1–2 days. From 3 to 6 days, the enzyme activity sharply increased to 2821U/g·Fw·h, and after 8 days, it continued to decrease to 1871U/g·Fw·h for two consecutive days. However, the enzyme activity was still significantly higher than that of the CK group and other treatment groups, indicating that the infection of *F. asiaticum* caused a substantial degree of stress to plants and produced a strong defense response. The FA + XJ-1 treatment group showed a slight decrease in enzyme activity from 1 to 2 days, followed by an initial increase and then a decrease from 4 to 10 days with little change. At 6 days, the highest enzyme activity reached 2596 U/g·Fw ·h. Within 10 days, overall enzyme activity was higher than that of the CK group and lower than that of the FA group. The PAL enzyme activity of the XJ-1 + FA group showed an overall trend similar to that of the FA + XJ-1 treatment group, showing an initial increase followed by a decrease; it reached its highest level of 2479U/g·Fw·h at 8days and decreased to 1668U/g·Fw·h at 10days. The enzyme activity of both treatment groups was higher than that of the CK and lower than that of the FA treatment group, indicating that both treatments can increase the disease resistance of maize plants. By increasing the expression of defense-related enzymes in maize plants, the pathogen’s potential to damage the plants is suppressed.

### Morphology of pathogenic fungal hyphae

**Fig. 6:**
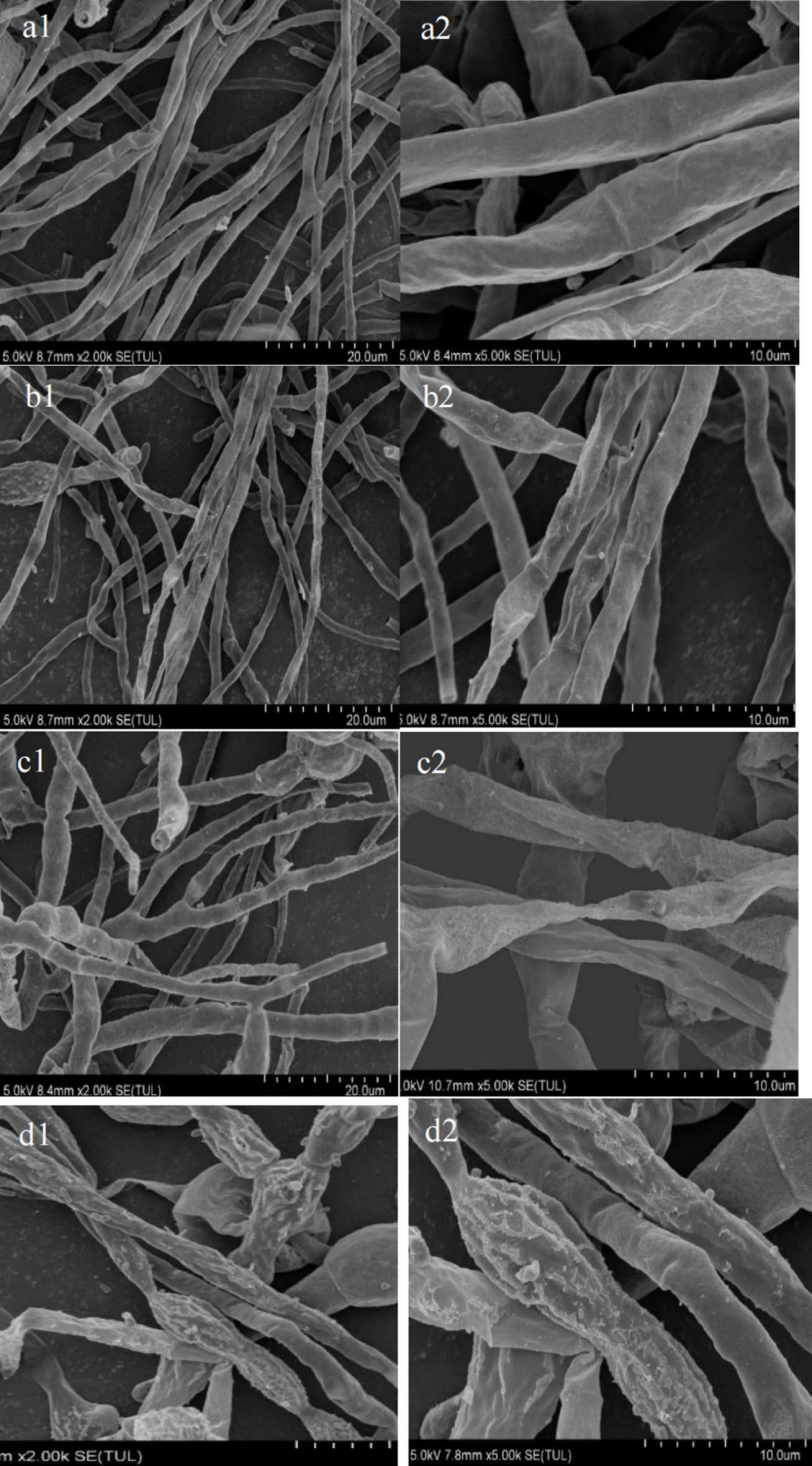
Effect of *Bacillus halotolerans* XJ-1 on the morphology of *F. asiaticum* determined by scanning electron microscopy. a1, control group (20 μm); a2, control group (10 μm); b1, treated group for 24 h (20 μm); b2, treated group for 24 h (10 μm); c1, treated group for 48 h (20 μm); c2, treated group for 48 h (10 μm); d1, treated group for 72 h (20 μm); d2, treated group for 72 h (10 μm).

Through scanning electron microscopy observation, Fig. 6 shows the effect of the fermentation supernatant of *B. halotolerans* XJ-1on the hyphae of *F. asiaticum.* Compared with the untreated control group, the morphological changes of the treated *F. asiaticum* hyphae were notable. In the control group, the hyphae of *F. asiaticum* showed a complete tissue structure, smooth surface, no obvious signs of shrinkage or breakage, and no depressions. The overall appearance was coarse and evenly distributed (Fig. 5a1, a2). However, after 24 h of treatment with the fermentation supernatant of strain XJ-1, *F. asiaticum* showed slightly withered and sunken hyphae, curved hyphae, and tangled knots (Fig. 5b1, b2). After 48 h of treatment, the phenomena of mycelial wilting and atrophy worsened, and the local area appeared in patches, indicating severe leakage of cell contents (Fig. 5c1, c2). After processing for 72 h, the mycelium showed abnormalities, local abnormal swelling, a significant increase in the secretion of surface attachments, irregular distortion, and obvious shrinkage (Fig. 5d1, d2).

Overall, compared with the smooth and round hyphae of the control group, the mycelia of each experimental group showed varying degrees of drying, wrinkling, twisting, and knotting. With the extension of treatment time, the mycelium of the treatment group in the later stage of the experiment also showed local abnormal swelling and deposition of surface attachments. Strain XJ-1 can secrete lytic substances that act on the surface of pathogenic mycelia, disrupting cell permeability and the balance between inside and outside the cell, causing the leakage of cell contents, blurring and dissolving of cell surface boundaries. Moreover, as the treatment time increased, the secretion of strain XJ-1 had a stronger destructive effect on the cell surface of *F. asiaticum*, with antimicrobial and even fungicidal effects becoming more obvious.

## Discussion

*Bacillus halotolerans* not only exhibits resistance activity against heavy metal pollution but also possesses broad-spectrum antimicrobial activity. Etemadzadeh et al.^30^ found that *B. halotolerans* DMG-7-2 demonstrates resistance to metals such as lead, calcium, chromium, nickel, copper, manganese, and mercury; can tolerate up to 12% salinity; and exhibits varying degrees of antibacterial activity against *Staphylococcus aureus*, *Pseudomonas aeruginosa*, *Escherichia coli*, and *Saccharomyces cerevisiae*. *B. halotolerans* also effectively controls tomato bacterial wilt and significantly reduces its occurrence.

Through pot experiments and laboratory analyses, this study systematically explored the maize stem base rot disease control and plant growth-promoting effects of *B. halotolerans* XJ-1. Strain XJ-1 significantly reduced the incidence and disease index of maize stem base rot, with protective and therapeutic control effects reaching 61.7% and 56.3%, respectively. Moreover, this strain significantly promoted the growth of maize plants, improving morphological (e.g., plant height, stem thickness, leaf area) and physiological (e.g., chlorophyll, soluble protein content) indicators. This study also found that inoculation of *B. halotolerans* XJ-1 and irrigation pesticide with carbendazim wettable powder could reduce the incidence rate and disease index of corn stalk rot; promote corn growth; and increase the total chlorophyll, soluble protein, and nitrate nitrogen contents of corn leaves. However, the relative control effect, total chlorophyll content, and soluble sugar content of the leaves treated with *B. halotolerans* XJ-1 were higher than those treated with the positive control (carbendazim), indicating that XJ-1 has a better therapeutic effect on corn stem rot and is more effective than the irrigation pesticide carbendazim wettable powder. These findings suggest that *B. halotolerans* XJ-1 can enhance plant disease resistance, thereby increasing yield, demonstrating its potential for application in micro fertilizers.

Chlorophyll is the material basis for photosynthesis in plants, playing an important role in the absorption, conversion, and utilization of light energy. In this study, the total chlorophyll content of maize leaves treated with *B. halotolerans* XJ-1 was significantly higher than that of the control group. When the pathogenic bacterium *F. asiaticum* was inoculated simultaneously, the total chlorophyll content of maize leaves treated with *Bacillus halotolerans* XJ-1 and irrigation pesticide carbendazim wettable powder was significantly higher than that treated with single inoculation of *F. asiaticum*.This indicates that the fermentation broth of *Bacillus halotolerans* XJ-1 can significantly increase the total chlorophyll content of maize leaves. Osmotic regulation is a physiological function of plants to resist external stress and ensure normal growth. Under adversity, plants will resist the damage of stress by accumulating osmoregulatory substances. Soluble sugars mainly participate in processes such as energy supply, signal transduction, osmotic regulation, and stress resistance in plants. Soluble proteins in plants have functions such as nitrogen storage, regulation of enzyme activity, improvement of stress resistance, participation in signal transduction, influencing growth and development, and promoting nutrient absorption, playing an important role in plant physiological growth. The results of this study indicate that the fermentation broth of *Bacillus halotolerans* XJ-1 significantly increased the soluble sugar and soluble protein content of maize leaves, enhancing the plant’s stress resistance. The absorption and utilization of nitrogen and phosphorus by plants directly affect their growth and development. Nitrate nitrogen is the most important nitrogen source for plants and can reflect the soil’s supply of nitrogen to plants. It is often used as a fertilizer indicator. As a major element required for plant growth and development, phosphorus participates in the composition and metabolism of compounds in plants, and determines crop yield and quality. Research by HUSL^31^ has shown that compared to CK, treatment with six endophytic bacteria can significantly increase the nitrate nitrogen and inorganic phosphorus content in Chuanxiong leaves. In this study, compared with the control, root irrigation with the fermentation broth of *Bacillus halotolerans* XJ-1 significantly increased the nitrate nitrogen and inorganic phosphorus content in corn leaves, which is consistent with previous research results.

This study further provides insight into the antifungal mechanism of the bacterial strain from the perspectives of modifying the ROS accumulation and activities of fungus defense-related enzymes. H_2_O_2_ plays an extremely important role in ROS metabolism: it can penetrate cells and cause damage to their structure and function, causing cell death^32^. H_2_O_2_ is the most common reactive oxygen molecule in living organisms and is mainly catalyzed and degraded by SOD, CAT, and POD. H_2_O_2_ can not only oxidize biological macromolecules such as nucleic acids and proteins within cells, causing damage to the cell membrane, but is also a key regulator in many oxidative stress responses. O_2_^−•^ has immune and signal transduction functions, but its excessive accumulation can damage cell membranes and the structure of biological macromolecules, leading to abnormal metabolism in the cells and tissues. •OH radicals can cause the oxidation of substances such as sugars, amino acids, proteins, and nucleic acids in tissues, leading to cell necrosis or mutation. Grant et al.^33^ found that O_2_^−^ and H_2_O_2_ have a direct inhibitory effect on bacterial growth. Gey et al.^34^ found that potato glycoside alkaloids can significantly increase the activity of *F. asiaticum*.The fermentation filtrate of XJ-1 significantly increased the contents of H_2_O_2_ and O_2_^−•^ and the clearance rate of •OH of *F. asiaticum*, while inhibiting the activities of defense enzymes such as SOD, POD, PPO, and PAL. This finding is consistent with previous studies showing that excessive accumulation of ROS can lead to cell membrane damage and metabolic disorders of pathogenic microorganisms, thereby inhibiting their growth^35–37^. Furthermore, *B. halotolerans* XJ-1 may directly destroy the cellular structure of pathogenic fungi by secreting antimicrobial substances (such as lipopeptide compounds)^38–40^, although determining its specific metabolites and target sites requires further study.

The active ingredients secreted by microorganisms with antimicrobial effects can target the cell wall, causing damage to the cell wall and weakening its protective barrier function, resulting in leakage of intracellular contents and achieving the effect of inhibiting pathogen growth^41^. In addition, these active ingredients can also reduce the surface tension of cells, form micropores on the cell membrane, and disrupt the surface structure of pathogens^42^. Some active ingredients can also interfere with the formation of the cell walls of pathogenic fungi, causing swelling and expansion of the hyphal tips, ultimately leading to pathogen death^43^. Jimnez et al.^44^ applied the fermentation supernatant of *Bacillus subtilis* Em7 to *Botrytis cinerea* and observed the impacts using scanning and transmission electron microscopy. Compared with the control group, the experimental group showed significant abnormalities in hyphal development, with some hyphae showing nodular swelling or contraction, an uneven and rough surface texture, uneven cell wall thickness, increased number of vacuoles inside the hyphae, cell wall rupture, tissue fluid leakage, and changes in cell membrane permeability. This indicated that the Em7 strain had a significant effect on inhibiting the growth of *Botrytis cinerea*. Similarly, our scanning electron microscopy observations showed that when the fermentation supernatant of strain XJ-1 was applied to *F. asiaticum* for 24 h, compared to the control group, the mycelium showed significant indentation and shrinkage, distorted and tangled morphology, and varying thickness; after 48 h of treatment, the substances inside the hyphae began to leak out, the degree of shrinkage intensified, and a small amount of substances were deposited on the surface of the hyphae; after 72 h, the mycelial morphology became irregular, with local swelling, increased surface attachments, and adhesion between hyphae. These observations confirmed that the secretion of strain XJ-1 has a destructive effect on the cell surface of *F. asiaticum* and can inhibit its growth.

Although this study confirms the potential of *Bacillus halotolerans* XJ-1 in the prevention and control of maize stem base rot, there some limitations that should be mentioned. For example, the plant growth-promoting effects are only based on pot experiments without corresponding field verification. Furthermore, the interaction mechanism between *Bacillus halotolerans* XJ-1 and the rhizosphere microbiota of maize has not yet been clarified. Future research can combine transcriptomics and metabolomics techniques to deeply analyze the molecular mechanism underlying the antifungal effects of strain XJ-1 and optimize its field application conditions to promote its popularization in actual agricultural production.

## Conclusion

In this study, through pot experiments and laboratory measurements, the pathogen control effect and plant growth-promoting effect of *Bacillus halotolerans* XJ-1 on maize stem base rot were systematically evaluated. Strain XJ-1 showed significant preventive and therapeutic effects on maize stem base rot, reaching 61.7% and 56.3%, respectively. Moreover, strain XJ-1 could significantly promote the growth of maize plants; increase morphological indicators such as plant height, stem thickness, and leaf area; and increase the contents of physiological indicators such as chlorophyll, soluble protein, and soluble sugar in the leaves. Strain XJ-1 appears to inhibit the growth of pathogenic bacteria by increasing the ROS content and hydroxyl radical-scavenging rate of *F. asiaticum*, while reducing the activities of defense enzymes such as SOD, POD, PPO, and PAL. Therefore, strain XJ-1 shows broad application prospects in the biological control of maize stem base rot and crop improvement, providing new ideas and theoretical basis for the development of green agriculture.

## Data Availability

Data is provided within the manuscript.

## Declarations

### Statement

Experimental research and field studies on plants in this research comply with Policy Statement on Research Involving Species at Risk of Extinction and the Convention on the Trade in Endangered Species of Wild Fauna and Flora.
